# Starr: Simple Tiling ARRay analysis of Affymetrix ChIP-chip data

**DOI:** 10.1186/1471-2105-11-194

**Published:** 2010-04-17

**Authors:** Benedikt Zacher, Pei Fen Kuan, Achim Tresch

**Affiliations:** 1 Gene Center, Department of Chemistry and Biochemistry, Ludwig-Maximilians-University of Munich, Feodor-Lynen-Str. 25, D-81377 Munich, Germany; 2 Department of Biostatistics and Lineberger Comprehensive Cancer Center, University of North Carolina at Chapel Hill, Chapel Hill, NC 27599, USA

## Abstract

**Background:**

Chromatin immunoprecipitation combined with DNA microarrays (ChIP-chip) is an assay used for investigating DNA-protein-binding or post-translational chromatin/histone modifications. As with all high-throughput technologies, it requires thorough bioinformatic processing of the data for which there is no standard yet. The primary goal is to reliably identify and localize genomic regions that bind a specific protein. Further investigation compares binding profiles of functionally related proteins, or binding profiles of the same proteins in different genetic backgrounds or experimental conditions. Ultimately, the goal is to gain a mechanistic understanding of the effects of DNA binding events on gene expression.

**Results:**

We present a free, open-source **R**/Bioconductor package *Starr *that facilitates comparative analysis of ChIP-chip data across experiments and across different microarray platforms. The package provides functions for data import, quality assessment, data visualization and exploration. *Starr *includes high-level analysis tools such as the alignment of ChIP signals along annotated features, correlation analysis of ChIP signals with complementary genomic data, peak-finding and comparative display of multiple clusters of binding profiles. It uses standard Bioconductor classes for maximum compatibility with other software. Moreover, *Starr *automatically updates microarray probe annotation files by a highly efficient remapping of microarray probe sequences to an arbitrary genome.

**Conclusion:**

*Starr *is an **R **package that covers the complete ChIP-chip workflow from data processing to binding pattern detection. It focuses on the high-level data analysis, e.g., it provides methods for the integration and combined statistical analysis of binding profiles and complementary functional genomics data. *Starr *enables systematic assessment of binding behaviour for groups of genes that are alingned along arbitrary genomic features.

## Background

Chromatin-ImmunoPrecipitation on chip (ChIP-chip) is a technique for identifying Protein-DNA interactions. For this purpose, the chromatin is bound to the protein of interest, then trimmed to yield a protein-bound fraction of DNA. The protein-bound fraction of DNA is then immunoprecipitated with a protein-specific antibody and hybridized to tiling microarrays [1]. The complex experimental procedure and the high dimensionality of the output data require thorough bioinformatical analyses which assess the quality of the experiments and ensures the reliability of the results [2,3]. The practical need for a ChIP-chip analysis tool has led to the development of either GUI-based or command line-oriented software (see [4,5], and [6,7], respectively). We favor the command line solution, which has been realized in our software, because virtually every ChIP-chip experiment requires flexible adaptations to its individual design as well as customized methods to test the hypotheses under investigation.

## Implementation

We present the open-source software package *Starr*, which is available as part of the open source Bioconductor project [8]. It is an extension package for the programming language and statistical environment **R **[9]. *Starr *facilitates analysis of ChIP-chip data, with particular but not exclusive support of the Affymetrix™ microarray platform. Its functionality comprises remapping of probe sequences to the genome, data import, quality assessment, and visual data exploration. *Starr *provides new high level analysis tools, e.g., the alignment of ChIP signals along annotated gene features, and combined analysis of the ChIP signals and complementary gene expression measurements. It uses the standard microarray data structures of Bioconductor, thus building on and fully exploiting the package *Ringo *[6]. The sequence mapping algorithm and some functions for peak finding are implemented in C to increase computation speed. The mapping of the probes to the position of the genome are stored in an object of the Bioconductor class *probeAnno*. Intensity measurements from the ChIP experiments are stored in an *ExpressionSet *object, which makes the results of *Starr *accessible to all other **R **packages that operate on these common classes.

## Results

**Figure 1:**
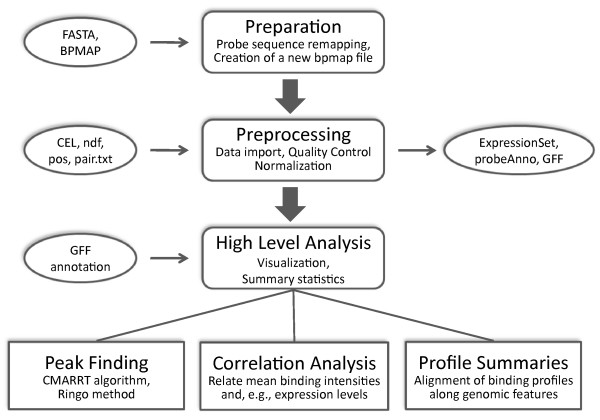
**Workflow**. Typical workflow of a ChIP-chip analysis in *Starr*, together with the utilized input resp. output files and data structures.

Figure 1 shows the typical workflow of a ChIP-chip analysis in *Starr*, together with the utilized input resp. output files and data structures. Our results description runs along the lines of this workflow, highlighting the novel features provided by *Starr*.

### Preparation

Sometimes a remapping of reporters to the genome may be necessary. This prevents probe sequences matching to either none or multiple sites of a current genome sequence, which might happen in the case of a probe annotation file being built upon an outdated version of the target species' genome sequence. Mending these false matches is mandatory, because all subsequent steps rely on correct probe annotation. Until now, this task required external programs like xMAN [10] or Mummer [11], which can be inconvenient and time-consuming. *Starr *contains a novel update function for microarray probe annotation (bpmap) files. It implements the Aho-Corasick algorithm [12], which is designed for efficient string matching. Remapping all probe sequences with *Starr *takes only seconds for small genomes like yeast and minutes for larger genomes like human (see Table 1, Results were calculated on an Intel Core Duo E8600 3.33 GHz machine). The result is a corrected, ready-to-use bpmap file.

**Table 1:** Time for remapping of Affymetrix reporter sequences to a genome

array	time	#sequences	genome size (bp)
S. cerevisiae Tiling 1.0R	34 s	2 697 594	12 495 682
Drosophila Tiling 2.0R	1 min 16 s	2 907 359	122 653 977
Human Promoter 1.0R	14 min 22 s	4 315 643	3.3 * 10^9^

### Preprocessing

We facilitated data import as much as possible, since in our experience, this is a major obstacle for the widespread use of **R **packages in the field of ChIP-chip analysis. Data import from the microarray manufacturers Nimblegen and Agilent has already been implemented in *Ringo*, the Affymetrix array platform is covered by *Starr*. There are two kinds of files that must be known to *Starr*: the .bpmap file which contains the mapping of the reporter sequences to their physical position on the array, and the .cel files which contain the actual measurement values. All data, no matter from which platform, are stored in the common Bioconductor object *ExpressionSet*, which makes them accessible to a number of **R **packages operating on that data structure. The built-in import procedure of Starr furthermore automatically creates R objects containing additional annotation (probeAnno, phenoData, sequence information), which is indespensible for our purposes. There exist alternative import functions, e.g., in the packages AffyTiling, oligo or rMAT [13], but these do not extract all the information we need, and often they use a different format. Genomic annotation can either be read directly from a gff file or obtained via the biomaRt package [14].

It would be desirable to discuss the structure of cel and gff files and of the ExpressionSet/probeAnno classes at greater length, but this is beyond the scope of this paper. We refer to the vignette of the *Starr *package, which addresses these more technical aspects in detail.

**Figure 2:**
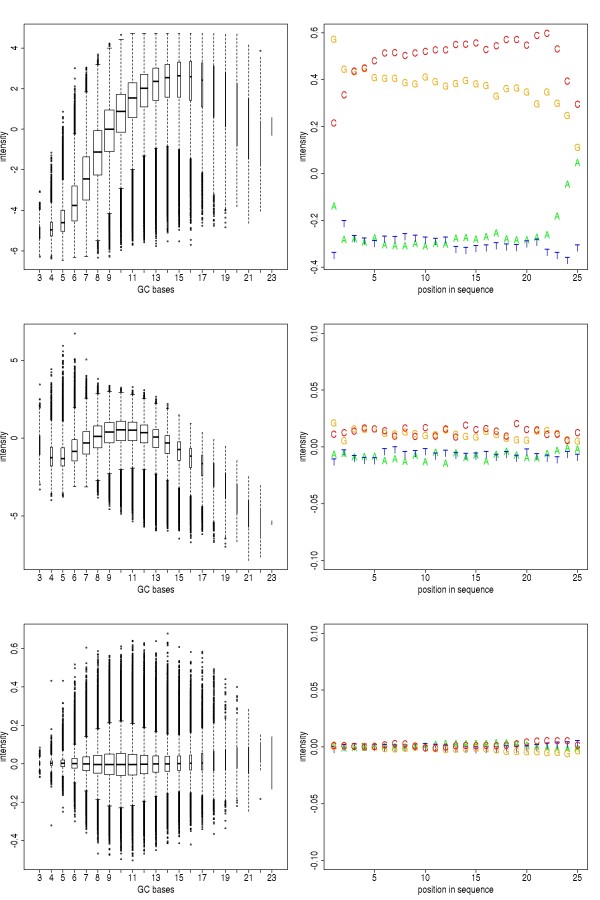
**Hybridization bias**. Sequence-specific dependency of reporter intensities before (top), after normalization using rMAT (middle) resp. after rank-percentile normalization and reference subtraction (bottom). Left column: Boxplots of probe intensity distributions. Probes are grouped according to the GC-content in their sequence. The median intensity increases with rising GC-content. Right column: Position-dependent mean probe intensity. Each letter corresponds to the mean intensity of all probes that contain the corresponding nucleotide in the respective position.

The obligatory second step in the analysis protocol is quality control. The complex experimental procedures of a ChIP-chip assay make errors almost inevitable. A special issue of Affymetrix oligo arrays is the intensity bias caused by the sequence-specific GC-content of the oligomer probes [2]. Sometimes this bias is not appropriately corrected due to improper normalization. Thus we included a new quality control plot routine for examining measurement bias and variation before and after normalization. *Starr *displays the average expression of probes as a function of their GC-content, and it calculates a position-specific bias of every nucleotide in each of the 25 positions within the probe (Figure 2). We used this visulization to check whether the MAT normalization method [15] does accurately correct for sequence-specific hybridization bias. We used the R implementation rMAT [13] of the algorithm for normalization. As is shown in Figure 2, it partly removes the systematic errors in the unnormalized data, yet the diagnostic plot reveals a strong residual bias in the processed data. We compared the performance of MAT to that of a normalization by a reference experiment. Such control measurements are obtained by either performing a mock immunoprecipitation, i.e. an immunoprecipitation which is designed to reflect unspecific antibody binding, or by simply digesting and processing genomic DNA of that organism. It turns out that a rank percentile normalization of experiment and reference, followed by a simple subtraction of the reference from the experiment measurements yields substantially better results than rMAT (bottom row of Figure 2). We therefore advise experimentalists to perform at least one control immunoprecipitation for normalization purposes. Moreover, *Starr *provides many other quality control plots like an in silico reconstruction of the physical array image to identify flawed regions on the array, or pairwise MA-plots, boxplots and heat-scatter plots to visualize pairwise dependencies within the dataset. For the purpose of bias removal (normalization), *Starr *interfaces the package *limma*. It also contains standard normalization methods like the loess normalization, or the median-rank-percentile normalization proposed by Buck and Lieb in 2004 [16]. For a comparison of ChIP-chip normalization methods, see recent publications [17-21].

### High-level analysis

We demonstrate the utility of *Starr *by applying it to a yeast RNA-Polymerase II (PolII for short) ChIP experiment. One of the most prominent purposes of ChIP experiments is the identification and localization of peaked binding events on the genome. Although, by virtue of compatibility, we can draw on the facilities of other peak detection algorithms like *Ringo *[6], ACME [22] or BAC [23], we implemented a novel algorithm - CMARRT - which was developed by P.F. Kuan [24] and performs well in practice. For further postprocessing of ChIP-enriched regions, we suggest the **R **package ChIPpeakAnno.

*Starr *provides functions for the visualization of a set of "profiles" (e.g. time series, or signal levels) along genomic positions. Our *profileplot *function relates to the conventional mean value plot like a box plot relates to an individual sample mean: Let the profiles be given as the rows of a samples × positions matrix that contains the respective signal of a sample at a given position. Instead of plotting a line for each profile (row of the matrix), the q-quantiles for each position (column of the matrix) are calculated, where q runs through a set of representative quantiles. Then for each q, the profile line of the q-quantiles is plotted. Color coding of the quantile profiles further aids the interpretation of the plot.

**Figure 3:**
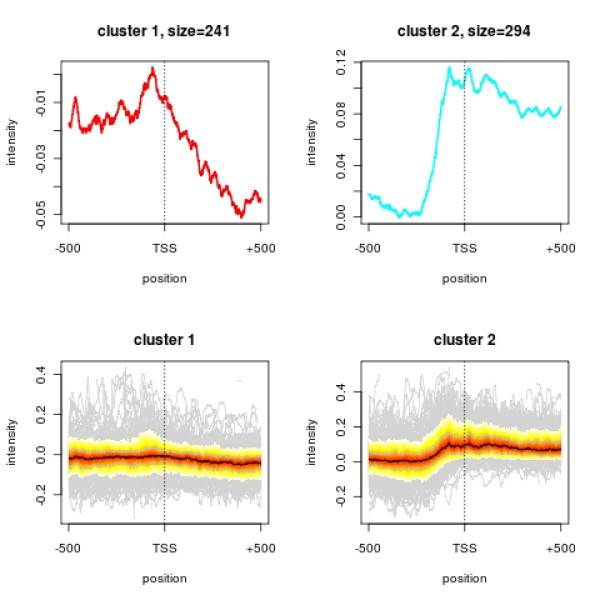
**PolII along the transcriptional start site**. Profiles of PolII occupancy of genes with low (least 20%) resp. high (top 10%) transcription rates (cluster 1 resp. cluster 2). The upper graphs show the mean occupancy calculated over each position along the transcription start site. The lower plots illustrate the same data, yet including the variance in the two clusters. The black line indicates the median profile of all features. The color gradient corresponds to quantiles (from 0.05 to 0.95), and the first and third quartiles are shown as grey lines. The light grey lines in the background show the profiles of individual "outlier" features.

Figure 3 shows a PolII ChIP experiment in which binding profiles have been aligned along the transcription start site for two different gene groups. The groups consist of the genes whose mRNA expression according to [25] ranges in the least 20% resp. the top 10% of all yeast genes (the cutoffs were chosen such that the number of genes having an annotated transcription start site was roughly the same within both groups). The common way of looking at the intensity profiles is to calculate and plot the mean intensity at each available position along the region of concern. Such an illustration however may be misleading, since it fails to capture the variability at each position. It is desirable to display this variability in order to assess whether a seemingly obvious alteration in DNA binding is significant or not. An instructive example is illustrated in Figure 3. The mean profile for genes with a low expression value shows an enrichment of PolII in the promotor region relative to the transcribed region. This could lead to interpretation that PolII is paused at the TSS of low expressed genes. However, the *profileplot *reveals that only very few genes with high binding intensities at the TSS determine the averaged profile.

**Figure 4:**
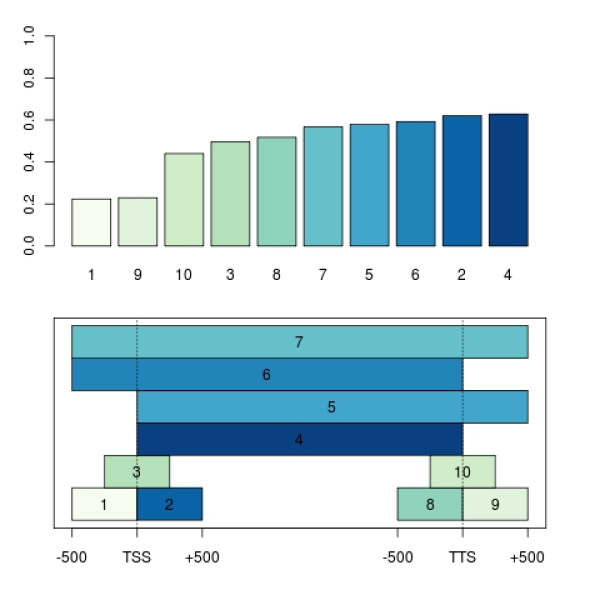
**Investigation of PolII-binding associated gene expression**. The upper panel shows the Pearson correlations of the mean PolII occupancy along different transcript regions and the corresponding mRNA expression levels. The lower panel marks the regions of interest relative to the transcription start site (TSS) and the transcription termination site (TTS).

Another useful high-level plot in *Starr *is the *correlationPlot*, which displays the correlation of a gene-related binding signal to its corresponding gene expression. Figure 4 shows a plot in which the mean PolII occupancy in various transcript regions of 2526 genes is compared to the corresponding mRNA expression. Each region is defined by its begin and end position relative to the transcription start and termination site (taken from [26]). The regions are plotted in the lower panel of Figure 4. For each region, the correlation between the vector of mean occupancies and the vector of gene expression values is calculated and shown in the upper panel. The correlation plot reveals that PolII occcupancy at the transcription start is not a good predictor of mRNA expression, but the mean occupancy of PolII in the elongation phase (region 4 in Figure 4) is. We expect that a more detailed analysis of particular gene groups, and a comparison of PolII profiles under different environmental conditions will yield valuable new insights.

## Conclusions

Apart from covering the standard processes of data acquisition and preprocessing, *Starr *is a Bioconductor package that offers a range of novel high-level tools that greatly enhance the exploration of ChIP-chip experiments. Those include functions like peak finding, summary visualization of gene groups, and correlation analysis with expression data. On the side of the low-level analysis, we implemented a convenient probe remapping algorithm that helps to keep annotation standards high. By relying on standard Bioconductor object classes, *Starr *can easily interface other Bioconductor packages. It therefore makes the full functionality of *Ringo *amenable to the analysis of Affymetrix tiling arrays. All functions and methods in the *Starr *package are well documented in help pages and in a vignette, which also contains a sample workflow in **R**. Altogether, *Starr *constitutes a powerful and comprehensive tool for tiling array analysis across established one- and two-color technologies like Affymetrix, Agilent and Nimblegen.

## Availability and requirements

The **R**-package *Starr *is available from the Bioconductor web site at http://www.bioconductor.org and runs on Linux, Mac OS and MS-Windows. It requires an installed version of **R **(version > = 2.10.0), which is freely available from the Comprehensive **R **Archive Network (CRAN) at http://cran.r-project.org, and other Bioconductor packages, namely Ringo, affy, affxparser, and vsn plus the CRAN package pspline and MASS. The easiest way to obtain the most recent version of the software, with all its dependencies, is to follow the instructions at http://www.bioconductor.org/download. Support is provided by the Bioconductor mailing list and the package maintainer. *Starr *is distributed under the terms of the Artistic License 2.0. An **R **script reproducing the entire results of this paper, together with the data files can be found in the supplements as Additional file 1, and on the website http://www.lmb.uni-muenchen.de/tresch/starr.html. ChIP-chip data of yeast PolII binding was published by Venters and Pugh in 2009 [27] and is available on array express under the accession number E-MEXP-1676. The gene expression data used here is available under accession number E-MEXP-2123. Transcription start and termination sites were obtained from David et al. [26].

## Authors' contributions

BZ implemented the *Starr *package and did the analysis. PFK contributed his implementation of the CMARRT algorithm. AT initiated and supervised the project. AT and BZ wrote the manuscript, and all authors approved of its final version.

## Supplementary Material

Additional file 1 **R script of high level analysis**. The **R **script that generates the plots of this paper is contained in the supplements. The data files that are needed to run the script can be downloaded either from public databases (Array Express/NCBI-GEO), or they can be obtained from http://www.lmb.uni-muenchen.de/tresch/starr.html. Click here for file
